# Batch Process Monitoring Based on Quality-Related Time-Batch 2D Evolution Information

**DOI:** 10.3390/s22062235

**Published:** 2022-03-14

**Authors:** Luping Zhao, Jiayang Yang

**Affiliations:** College of Information Science and Engineering, Northeastern University, Shenyang 110819, China; 20194179@stu.neu.edu.cn

**Keywords:** batch process, partial least squares, online monitoring, evolution information

## Abstract

This paper proposed a quality-related online monitoring strategy based on time and batch two-dimensional evolution information for batch processes. In the direction of time, considering the difference between each phase and the steady part and the transition part in the same phase, the change trend of the regression coefficient of the PLS model is used to divide each batch into phases, and each phase into parts. The phases, the steady parts, and the transition parts are finally distinguished and dealt with separately in the subsequent modeling process. In the batch direction, considering the slow time-varying characteristics of batch evolution, sliding windows are used to perform mode division by analyzing the evolution trend of the score matrix **T** in the PLS model on the base of phase division and within-phase part division. Finally, an online monitoring model that comprehensively considers the evolution information of time and batch is obtained. In a typical batch operation process, injection molding is used as an example for experimental analysis. The results show that the proposed algorithm takes advantage of mixing the time-batch two-dimensional evolution information. Compared with the traditional methods, the proposed method can overcome the shortcomings caused by the single dimension analysis and has better monitoring results.

## 1. Introduction

The batch process is an important mode of production in the modern manufacturing industry. It refers to the manufacturing process in which the input raw materials are transformed into one or a batch of desired products in a limited phase by predetermined procedures and repeated. Finally, more of the same products are obtained. In today’s society, market demand changes rapidly, and high value-added products are produced by batch processing, so the operation safety issue has aroused people’s attention [1,2,3,4].

It is expected that the batch process can be monitored efficiently; that is, the actual running state of the production process can be sensitively sensed, and whether the whole or part of the system is running properly can be analyzed. Early detection of abnormal production conditions affecting the quality of products; corresponding countermeasures can be given for different abnormal situations [5]; the monitoring of batch processes plays an important role in maintaining safety and ensuring the high quality of products. It can be divided into two aspects: condition monitoring and fault diagnosis. Condition monitoring [6] refers to the perception, analysis, and evaluation of the operating status of the production process, analysis based on the collected data, understanding of the historical situation and operating status of the system, fully considering the impact of external factors, including the environment, to judge whether it is normal and evaluating the operating level. Fault diagnosis [6] is used to analyze the detected abnormal state further, to understand the internal causes and influencing factors, and use the mining and understanding of historical faults and maintenance records. On the one hand, it analyzes and judges the existing faults; on the other hand, it forecasts the possible faults of the equipment in advance. Research on batch process monitoring has attracted the attention of many researchers. The production model of the batch process is different from that of continuous processes. The most significant difference between the two is that the products and process operating conditions of batch production change frequently. Batch processes are nonstationary, and their statistical properties change over time [7,8,9]. Data parsing methods [10,11,12,13], which extract information from process historical data and conduct modeling monitoring based on it, have become a hot topic in process monitoring research. Among them, principal component analysis (PCA) [14,15] and partial least squares (PLS) [16,17] have become the classical algorithms to study the characteristics of batch process data. Compared with qualitative knowledge such as expert experience, the data analysis method can be understood as a special knowledge-based method, which does not require an accurate systematic model and prior knowledge. The development of data parsing methods also provides theoretical guidance for process monitoring research. Data analysis methods have been developed and improved in recent years, and batch process monitoring and fault diagnosis technology based on data analysis have become a hot research topic.

Batch process evolution exists in both time and batch directions. Tracking batch process evolution is significant for process monitoring and quality prediction. In the time direction, multiple phases are a distinct character with the switching of operation procedures, and the characteristics of batch processes are significantly different in different phases. Based on multi-phases process monitoring, it can better reflect the change rule of time and highlight the local process’s characteristics, especially for the fault with small amplitude, can be timely identified. Lu et al. [18] and Zhao et al. [19] proposed a theoretical method for analyzing the change rule of nonstationary characteristics and multiphase characterization. They hold the following views on the multiphase batch process: They believe that the potential characteristics of batch operation processes change regularly with the changes in process operation or process mechanism characteristics. Additionally, the internal process operation mechanism changes can be inferred from the changes in the process statistical characteristics. The batch operation cycle is divided into several sub-phases by using the correlation between process variables or between process variables and quality variables. The correlation characteristics of variables in the same phase remain approximately the same. The most basic multiphase division method is the process mechanism method [20], which divides the operation phase by taking advantage of the actual mechanism changes of batch industrial process operation and relying on expert experience or existing knowledge. After the phases are divided, the model is established separately in different phases, and the state is monitored separately. However, for each phase in the batch process, its internal characteristics are not completely in a stable state. There are usually transition states during the phase shift [19]. In a transition state, the process-related characteristics tend to show gradual change. Based on the idea of division within a phase, Zhao et al. [21] separated the transition parts and the steady parts and established monitoring models, respectively, which further captured the evolution information in the time direction. 

In the batch direction, due to the influence of various factors, such as changes in raw materials and components, changes in the external environment, and requirements of different products, the operating state of the production process changes slowly, and the manufacturing process often transforms between different modes. This slowly changing phenomenon is very common in actual batch processes, which is different from faults, and it does not affect the process safety of batch operation. Therefore, this normal variation needs to be accurately distinguished from true faults during the modeling process. From a data analytic modeling perspective, the variety in the process variable data can map slow changes from batch to batch. Specifically, on the one hand, these slow time changes in actual production will lead to slow shifts of process variables, and further, these data changes can be transformed into the change trends of latent variables through the data analysis method, which can be clearly observed. On the other hand, using data analysis, the evolution information of the process variables is used to infer the changes of the production state in the batch direction so as to complete the mode division and obtain a more accurate offline monitoring model. For the evolution law in the batch direction, researchers have proposed many mature algorithms, such as the adaptive PCA algorithm proposed by Li et al. [22] and the sliding window method proposed by Zhao et al. [23]. The authors considered the variation information of working conditions between batches and used the characteristics of slow time-varying in batch direction to conduct mode division.

However, the above studies are all one-dimensional evolution studies based on the direction of time or batch. How to consider the variation information in both the direction of batch and time is still a significant problem to be solved. Based on previous studies, in this paper, both the piece of inter-batch evolution information and the piece of inner-batch evolution information are comprehensively considered to divide the batch process into phases, the steady part and transition part within phases, and modes for the first time, and then quality regression models are built for process monitoring. First of all, in the time direction, the regression coefficient ***β***, calculated by PLS, is used to distinguish phases, transition parts, and stable parts in the same phase, thereby realizing the classification of the dimension of time. In the batch direction, the influence of the sliding window parameters on the modeling results is further studied, and the operation law of the sliding window is revealed. This paper innovatively studied the evolution of score matrix **T** and load matrix **P** calculated by PLS in the direction of the batch. We proposed a simple and effective mode division method; that is, the variance information of score matrix **T** obtained by the PLS algorithm is analyzed, and the deviation between the current window and the first window is used to divide the mode. Moreover, this mode division strategy is innovatively based on the within-phase division. Therefore, both pieces of the time and batch evolution information are captured for accurate model establishment. In the final online monitoring strategy, time information is used to identify the steady part and transition part data with differences. The sliding windows in the batch direction are established to identify the modes. This operation considers the difference information of different operation phases and within-phase parts in the time direction. It considers the difference in information caused by slow time-varying in the batch direction. This scheme may obtain more comprehensive operating mode change information and provide a multi-dimensional batch process of detecting state online monitoring and fault diagnosis strategy. 

The content of this paper covers the following aspects: the second part introduces the operation process of the PLS algorithm; The third part introduces the data modeling method using time-batch two-dimensional evolution information and puts forward the strategy of online monitoring. In the fourth part, the injection molding process is taken as an example to verify the validity of the proposed method. Finally, this paper is summarized.

## 2. Methodology

### 2.1. Partial Least Squares Method (PLS)

For a typical batch process, the process data is stored in a three-dimensional matrix, $X\left(I\times J_{x}\times K\right)$, where *I* represents the number of batches, $J_{x}$ represents the number of process variables, and K represents the number of sampling moments in a batch. Meanwhile, in the batch operation, the quality data is obtained and is stored in the quality matrix, $Y\left(I\times J_{y}\right)$, where $J_{y}$ represents the number of quality variables. For the case of only one quality variable, the quality matrix can be simplified by a one-dimensional vector. The partial least square method (PLS) is a classical method for online monitoring, fault diagnosis, and quality prediction of batch processes. It can carry out regression modeling and simplify the structure of data and analyze the correlation between two groups of variables [16]. Using the PLS algorithm, the correlation between process variables and quality variables can be transformed into shallow variable space for research. Its main formula is as follows:

(1) $$\begin{array}{c} X=TP^{T}+E=\sum_{a=1}^{A}t_{a}{p_{a}}^{T}+E\\ Y=UQ^{T}+F=\sum_{a=1}^{A}u_{a}{q_{a}}^{T}+F \end{array}$$

The above formula can be interpreted as the principal component decomposition of **X** and **Y** matrices. **T** and **P** represent the score matrix and load matrix of the principal component of the process variable, **X**, respectively. **U** and **Q** represent the score matrix and load matrix of the principal component of the quality variable, **Y**, respectively. In contrast, **E** and **F** represent the residual matrix of the principal component model.

The regression model based on PLS can be expressed as follows:

(2) Y=XB

where **B** is the regression coefficient matrix. If there is only one quality variable, that is, the mass matrix is a one-dimensional vector, the prediction model can be simplified into the following form:

(3) y=Xβ

It should be pointed out that both **X** and **Y** in the formula are two-dimensional matrices. Therefore, before using PLS for modeling, it is necessary to reduce its dimension first. In previous studies, there are mainly two treatments when using PLS to deal with issues related to batch processes. One is to directly extract the data of each sampling time for PLS modeling, which is called time slice [21] and denoted as $X_{k}\left(I\times J_{x}\right)$. The other is to take the mean value matrix along the direction of time [21], whose formula is shown below.

(4) $${\bar{X}}_{m}=\frac{1}{K_{m}}\sum_{k=1}^{K_{m}}X_{k}$$

where $K_{m}$ represents the number of time slices considered in the specific modeling situation, which will be given in detail in Section 2.3. Then the data are normalized, and the data in the process variable and quality variable matrix are converted to the data with the mean value of 0 and variance of 1. Finally, the data can be substituted into the PLS formula. This paper uses the PLS algorithm to build offline models and monitor the batch process online. The commonly used statistics are Hotelling-*T*^2^ statistics and square prediction error *SPE* statistics. Hotelling-*T*^2^ statistics reflect the deviation degree of latent variables from the established model in amplitude and process data development trend. *SPE* describes the deviation degree of the measured value of the input variable from the latent variable space in the batch process [20]. The specific modeling process’s specific calculation methods are different, which will be given in the later introduction.

### 2.2. Model Based on Time-Batch 2D Evolution Information

**Figure 1:**
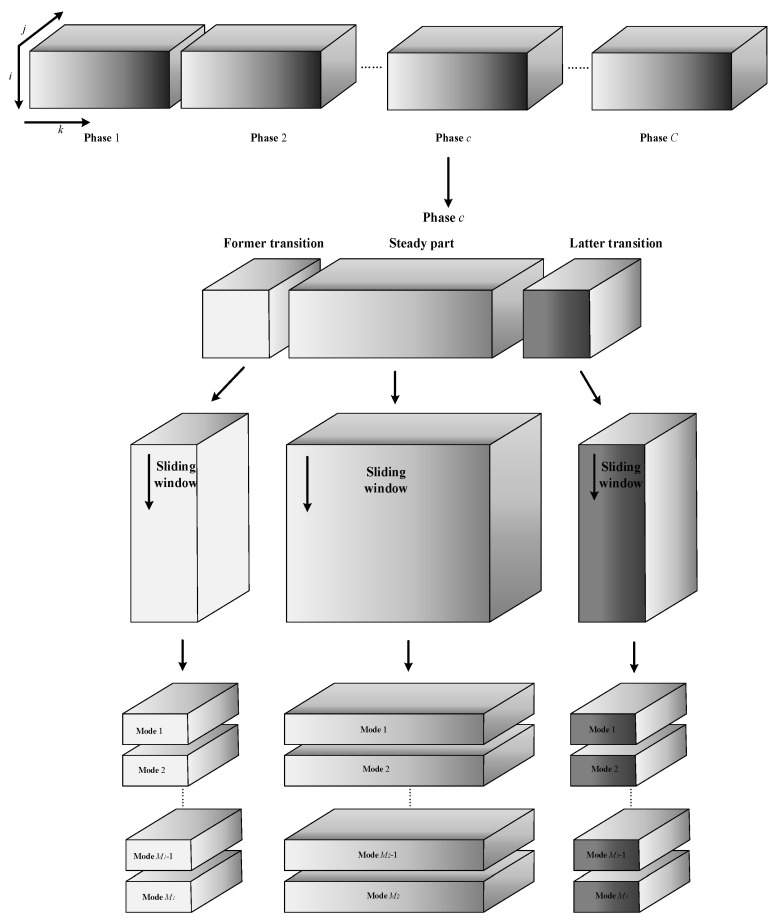
Schematic diagram of the overall offline monitoring model establishment.

In batch processes, data is critical in both the time and batch directions. Therefore, it is necessary to consider the evolution of information in these two directions to obtain a more accurate monitoring model. In the time direction, since different phases in the batch process will be different, and different parts of each phase will be different, the strategy of phase division and inner-phase division is taken to distinguish different phases and parts in each phase to achieve a more accurate modeling effect. The PLS algorithm is used to separate the phases, the steady part and the transition part within phases using the regression coefficient β. In the direction of the batch, due to the existence of slow time-varying, different operating modes need to be identified to establish a more well-matched monitoring model. A sliding window is introduced to cluster the batch into multiple modes. Finally, these two pieces of evolution information in the two directions of time and batch are combined to build an offline model for online monitoring. The establishment of the overall offline monitoring model is shown in Figure 1. The following is a specific introduction.

#### 2.2.1. In-Phase Analysis in Time Direction

Firstly, the strategy of phase division and inner-phase division is proposed to distinguish different phases and different parts in each phase. Using batch knowledge, each batch is divided into *C* phases. According to the form of time slices of time *k*, the PLS algorithm is used to calculate the regression coefficient vector β of each time slice, and the changing trend of its value in the direction of *K* is studied [24]. By calculating the gradient, data in the whole phase can be divided into the transition steady part and transition part. Considering the discreteness of data, the gradient of β is expressed in the form of difference. The calculation formula is as follows:

(5) $$diff_{\beta }\left(k\right)=\left|\beta_{k+1}-\beta_{k}\right|$$

When the calculated value, $diff_{\beta }$, exceeds the preset threshold, the moment *k* is judged to be a transition part; otherwise, it is considered a steady part. However, if the difference operation is carried out only for the previous or next sampling time, it will have the risk of misclassification because it is very sensitive to noisy data [25]. Therefore, the following formula is introduced here to modify the gradient index:

(6) $${\bar{diff}}_{\beta }\left(k\right)=\frac{1}{m}\sqrt{\sum_{K=k}^{k+m-1}diff_{\beta }{\left(K\right)}^{2}}$$

In the formula, *m* is the parameter that can be set. For each phase, appropriate thresholds are taken to divide the steady part and transition part. When the threshold value is selected, if the threshold value is small, there will be fewer moments contained in the stable part, and correspondingly, there will be more moments in the transition part. Otherwise, if the threshold value is large, more moments will be contained in the stable part and fewer moments in the transition part. According to the previous research [24], if the threshold is strictly set, the proportion of steady part data in the whole phase is only about half. Therefore, in the direction of time *K*, it is necessary and meaningful to extract steady part and transition part data separately and conduct modeling, respectively.

#### 2.2.2. Establishment of Sliding Windows and Division of Modes in Batch Direction

**Figure 2:**
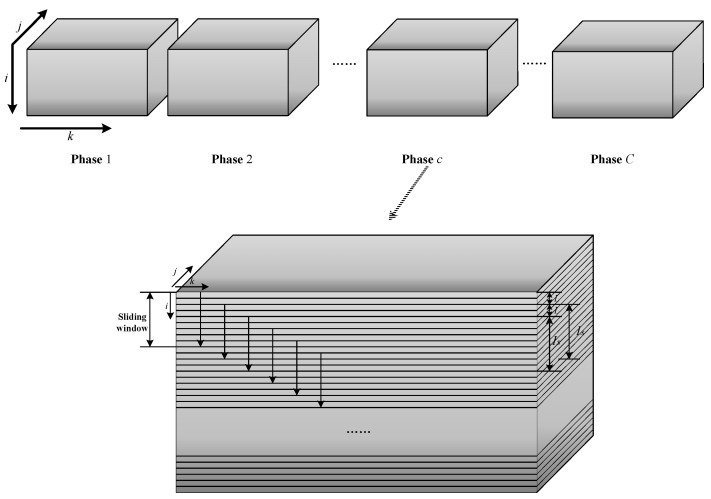
Development of sliding window in batch direction.

After considering the evolution in the time direction, the model is also refined from the perspective of batch evolution. Due to equipment aging and other reasons, batch processes have slow time-varying characteristics in the batch direction. Therefore, it is necessary to study the variation of data in the direction of the batch. Zhao [23] proposed the concept of a generalized sliding window in the batch direction. Figure 2 is a schematic diagram of the sliding window. After the inner-phase division is completed, process data in each phase is a three-dimensional matrix, $X_{c}\left(I\times J_{x}\times K_{c}\right)$, where *I* represents the number of batches in the *c*-th phase, $J_{x}$ represents the number of process variables, and $K_{c}$ represents the number of sampling moments in the *c*-th phase. The weight data is a two-dimensional matrix, $Y_{c}\left(I\times J_{y}\right)$, where $J_{y}$ represents the number of quality variables. The first window is established using the data of the first $I_{w}$ batch, $X_{c,1}\left(I_{w}\times J_{x}\times K_{c}\right)$, and the corresponding quality data is $Y_{c,1}\left(I_{w}\times J_{y}\right)$. This window only contains the information of the first to $I_{w}$-th batches. Then, the window is moved down by *L* batches; that is, the second window is formed using the data of the (L+1)th to $\left(L+I_{w}\right)$th batches. Following the steps described above, a new window is defined every time *L* batches are moved down until all batches are utilized to build the window. *L* is advised to be 1 [26]. Finally, $S=I-I_{w}$ windows are obtained, denoted as $X_{c,s}\left(I_{w}\times J_{x}\times K_{c}\right)$ respectively, and the corresponding quality data is $Y_{c,s}\left(I_{w}\times J_{y}\right),s=1,2,\;\cdots ,\;S$. These sliding windows are built to achieve mode division in the direction of the batch, and there are some steps before the mode division. First, the time slices in $X_{c,s}\left(I_{w}\times J_{x}\times K_{c}\right)$ are normalized around the center of data of the first window to obtain the changing trend of data along the batch direction. Next, the variation rule of the score matrix **T** after PLS modeling in the direction of the batch will be studied.

The 3D data matrix, $X_{c,s}\left(I_{w}\times J_{x}\times K_{c}\right)$, is expanded into a 2D matrix, ${\underline{X}}_{c,s}\left(I_{w}\times J_{x}K_{c}\right)$, along the batch direction to obtain process data for each batch. Substitute variable data of each window into the PLS formula, and the corresponding score matrix $T_{c,s}$ of each window can be obtained. Then, the following indicators are used to calculate the changing trend in the direction of batch windows:

(7) $$\begin{array}{c} Ratio_{s,var}=\frac{var\left(T_{c,s}\left(:,i\right)\right)}{var\left(T_{c,1}\left(:,i\right)\right)},i=1,2,\;\cdots ,\;R_{c,s}\\ Ratio_{s,mean}=\frac{mean\left(T_{c,s}\left(;,i\right)\right)}{mean\left(T_{c,1}\left(:,i\right)\right)},i=1,2,\;\cdots ,\;R_{c,s} \end{array}$$

For the *s*-th window, if the calculated *Ratio* value is larger, it means that the data in this window deviates more significantly from the data in the first window. For the first window, the calculated *Ratio* value is 1. According to previous studies [25], the first principal component, namely the principal component with the largest variance contribution rate, contains the largest amount of information and can reflect the dynamic characteristics of original data to a large extent. To simplify the algorithm, only the first principal component information is taken for research; that is, the parameter *i* is set to 1. Both the variation of variance of the score matrix and the variation of the mean value of the score matrix in the batch direction, reflected in the evolution of $Ratio_{s,var}$ and $Ratio_{s,mean}$, are studied. The window length will affect the change result of the *Ratio*. The shorter the window length, the better it can reflect the local data changes in the batch direction. However, if the window length is too short, the data trend will be unstable, and it is difficult to capture the actual evolution trend of variables in the batch direction. The longer the window length, the smoother the calculated index, but some small local changes in the batch direction will be lost. Meanwhile, according to the modeling experience in multivariate statistical regression [27], the window length should be set to 2–3 times the number of process variables. 

**Figure 3:**
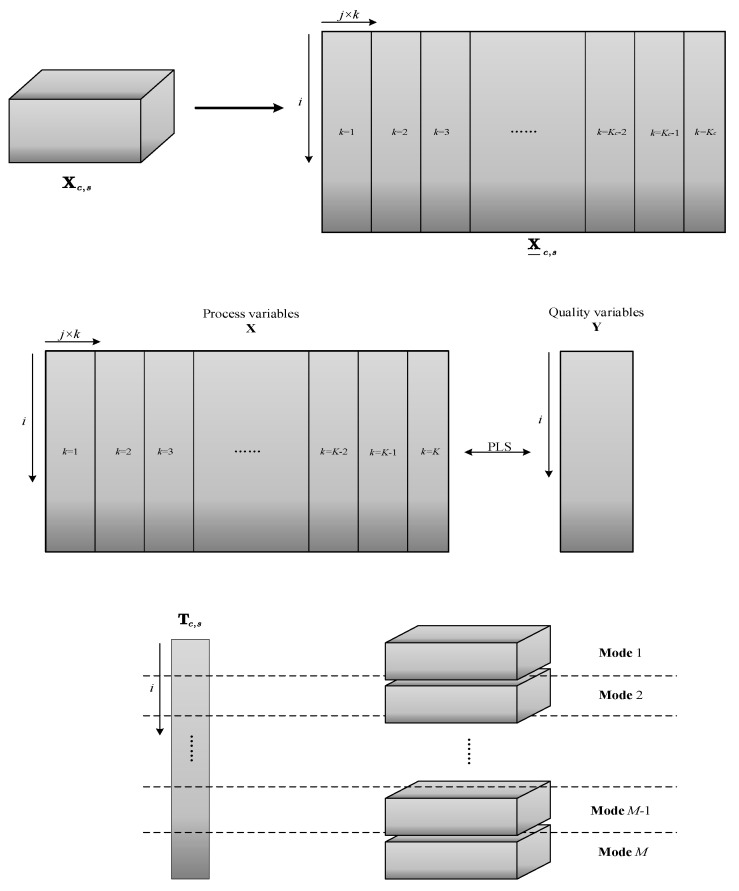
Mode division strategy in batch direction.

Similarly, the variation trend of the load matrix $P_{c,s}$ is studied with window sliding. It is described by means and variance data of its first principal component. The operation process is the same as the **T** index. After that, the **T** matrix or **P** matrix is used for mode division in batch direction. A schematic diagram of this process is shown in Figure 3.

**Figure 4:**
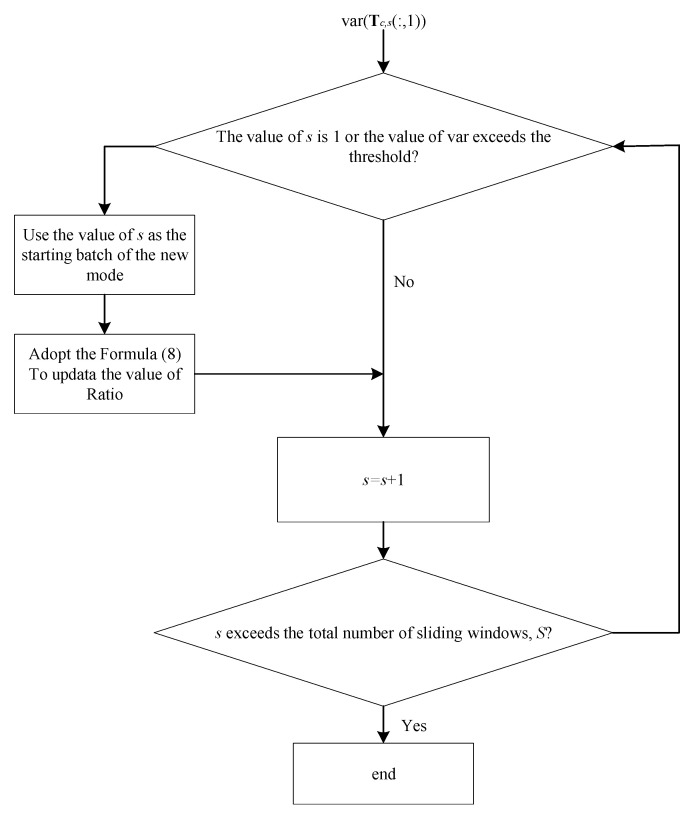
The flow diagram of iteration of the value of *Ratio*.

Zhao et al. [23] applied the concept of mode into the sliding window. They divided variable data **X** into multiple modes along the direction of the sliding window, but the classification method is relatively complicated. In this paper, a simplified algorithm is proposed, efficiently dividing modes in a shorter time and achieving the same effect. In the batch process, due to the existence of slow time variation, the correlation information of variables will gradually deviate from the initial state. The variance or mean characterizes slow changes in these correlated variables, thereby dividing the modes. Take the variance index of the score matrix **T** as an example; its algorithm process is as follows: for $var\left(T_{c,s}\left(:,1\right)\right)$, the number 1 represents the first principal component, divided it by $var\left(T_{c,1}\left(:,1\right)\right)$, the first data of the sliding window, and obtain $Ratio_{s,var,1},s=1,2,\;\cdots ,\;S$. Then observe the Ratio data for the second and subsequent windows, $Ratio_{s,var,1},s=2,3,\;\cdots ,\;S$. If the value is not more than the set threshold, the windows keep sliding down, and all windows whose *Ratio* data does not exceed the threshold are divided into the same mode; If the value exceeds the set threshold when sliding down to the *se*-th window, it is considered to be a new mode, $Ratio_{s,var,1}$ is divided by $Ratio_{se,var,1}$ that under the window *se*, and the *Ratio* is updated into $Ratio_{s,var,2},s=1,2,\;\cdots ,\;S$. Repeat the above operations until the end of the sliding window sliding. The process of the iteration of the *Ratio* is shown in Figure 4. The iteration formula of each *Ratio* is as follows:

(8) $$Ratio_{s,var,new}=\frac{Ratio_{s,var,old}}{Ratio_{se,var,old}},s=1,2,\;\cdots ,\;S$$

Actually, new=old+1. Similarly, there is the following iteration formula:

(9) $$Ratio_{s,mean,new}=\frac{Ratio_{s,mean,old}}{Ratio_{se,mean,old}}$$

In addition, there are two points to be noted. First, the sliding window will only slide to which starting with the first batch of $I_{w}-L+1$, and apparently after that can no longer slide. According to the knowledge of batch processes, the evolution information in the last few batches is not big. It gradually flattens, so the last remaining batches can be assumed to belong to the last mode divided before forming a mode directly. Second, in some cases, the same modality may contain only a small number of batches, resulting in a small amount of data, which reduces the robustness of the PLS algorithm. Therefore, the solution proposed here is, based on modeling experience, to add succeeding batches in this modality. After the batches are added, the modeling is carried out. This is done because it is proposed in the first point that the first batch of each window is used as the representative batch, but, in addition to the first batch in a window, there is also information on the following *L* − 1 batches. Therefore, the above-mentioned method of supplementing batches backward can make the amount of modeling data meet the requirements and strengthen the ability of the modal representation of batches.

A complete offline modeling strategy is proposed after considering the evolution information in time and batch directions. First, the gradient information of the regression coefficient matrix of the PLS model, $diff_{\beta }$, is used in the time direction to realize the division of phases and inner-phases and obtain phases, steady parts and transition parts. For the steady parts and transition parts in each phase, use a sliding window to finish the mode division in the batch direction. The final offline model contains both the evolution information in the time direction and the evolution information in the batch direction.

### 2.3. Online Monitoring Strategy

The content of the above two subsections respectively introduces the modeling based on the evolution information of the time and batch direction. In online monitoring, it is also necessary to fuse the evolution information of the two dimensions. For an online sample point of a batch, it is necessary to identify which phase it belongs to, whether it is in a steady-state or a transitional state and which mode it belongs to. Offline data that exactly matches the above information are used to model. It is worth pointing out that the calculation of statistics and detection limits is different for the steady parts and the transition parts in the online monitoring strategy. For steady part data, due to its small variation range with time, only one model needs to be established in the average sense. Due to its strong instability, transition part data modeling in the form of a time slice is needed. 

**Figure 5:**
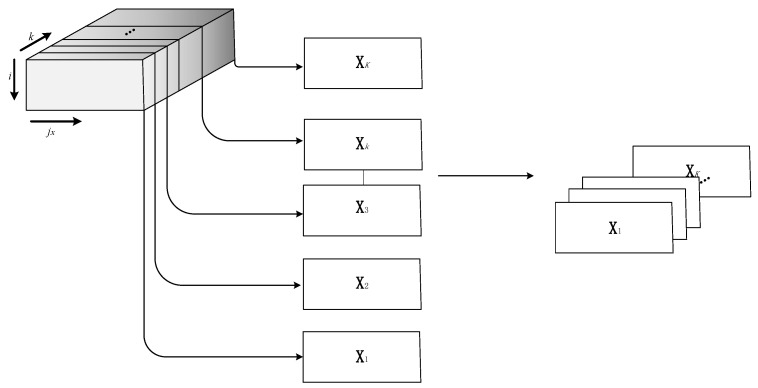
The extraction process of the time slices.

The specific algorithm flow is as follows. According to the results of division within the above phases, each phase is divided into former transition, latter transition, and steady part according to time. During online monitoring, the data at each time in the batch is judged to which phase it belongs to and then to determine whether it belongs to the transition part or steady part. Finally, a sliding window is built, and which mode the batch belongs to is determined. For the online data just collected, first, the number of the batch to which it belongs is identified. Its corresponding sliding window is found, and the strategy of mode division proposed in Section 2.2.2 is used to find the mode of the window. Finally, the data of the online batch is considered to belong to the mode acquired. For steady part data, the mean method is used for offline monitoring modeling; for transition part data, the time slice method is used for offline monitoring modeling. The extraction process of the time slice is shown in Figure 5. The time slice method is to extract the data matrix at each time to calculate the statistics and finally combine them to realize online monitoring. Unlike the mean method, which takes the mean value of all time slices, this method considers the differences of data at each time and is well suited for online monitoring of transition parts.

As mentioned above, for the stable part and the transition part, the online modeling strategies are different. The following are the specific introductions of the two.

The steady part modeling steps of stage *c* and mode *m* are as follows: Take the mean value of the three-dimensional process variable data matrix $X_{c,m,st}$ along the time direction:

(10) $${\bar{X}}_{c,m,st}=\sum_{k\in k_{st}}X_{c,m,st,k}/K$$

$X_{c,m,st,k}$ is the data matrix at the *k*-th time of the steady part. Substitute ${\bar{X}}_{c,m,st}$ into the PLS model:

(11) $$\begin{array}{c} {\bar{X}}_{c,m,st}=T_{c,m,st}{P_{c,m,st}}^{T}+E_{c,m,st}=\sum_{a=1}^{A}t_{c,m,st,a}p_{c,m,st,a}^{T}+E_{c,m,st}\\ {\bar{Y}}_{c,m,st}=U_{c,m,st}{Q_{c,m,st}}^{T}+F_{c,m,st}=\sum_{a=1}^{A}u_{c,m,st,a}q_{c,m,st,a}^{T}+F_{c,m,st} \end{array}$$

The specific meaning of each variable in the formula is explained in Section 2.2. Then, the monitoring statistics are calculated:

(12) $$\begin{array}{c} T_{c,m,st}^{2}=x_{c,m,st}^{T}R_{c,m,st}{\left(\frac{{T_{c,m,st}}^{T}T_{c,m,st}}{I_{m}-1}\right)}^{-1}{R_{c,m,st}}^{T}x_{c,m,st}\\ SPE_{c,m,st}=\|{\tilde{x}}_{c,m,st}{\|}^{2}=\|\left(I_{J_{x}}-P_{c,m,st}{R_{c,m,st}}^{T}\right)x_{c,m,st}{\|}^{2} \end{array}$$

where ${\tilde{x}}_{c,m,st}$ is the residual matrix, and $R_{c,m,st}$ is calculated as follows:

(13) $$R_{c,m,st}=W_{c,m,st}{\left(P_{c,m,st}^{T}W_{c,m,st}\right)}^{-1}$$

$W_{c,m,st}$ is the mass matrix, the specific calculation details are in Reference [28]. The corresponding control limits are as follows:

(14) $$\begin{array}{c} T_{c,m,st,\alpha }^{2}=\frac{H\left(I^{2}-1\right)}{I\left(I-H\right)}F_{c,m,st,\alpha }\left(H,I-H\right)\\ SPE_{c,m,st,\alpha }=g_{c,m,st}\chi_{c,m,st,h,\alpha }^{2} \end{array}$$

where $F_{c,m,st,\alpha }\left(H,I-H\right)$ means the ***F*** distribution with the confidence level α and the degrees of freedom *H* and *I* − *H*, and *H* refers to the number of retained latent variables; $g_{c,m,st}\chi_{c,m,st,h,\alpha }^{2}$ means the $\chi^{2}$ distribution with the confidence level α and the proportional coefficient $g_{c,m,st}=s_{c,m,st}/2\mu_{c,m,st}$; $h_{c,m,st}=2\mu_{c,m,st}^{2}/s_{c,m,st}$; μ refers to the mean value of *SPE*; s is the variance of *SPE*.

After obtaining the data to monitor, $x_{c,m,st,k,new}$, calculate the online *T*^2^ data and the online *SPE* data. The calculation formula is as follows:

(15) $$\begin{array}{c} T_{c,m,st,k}^{2}=x_{c,m,st,k,new}^{T}R_{c,m,st}{\left(\frac{{T_{c,m,st}}^{T}T_{c,m,st}}{I_{m}-1}\right)}^{-1}{R_{c,m,st}}^{T}x_{c,m,st,k,new}\\ SPE_{c,m,st,k}=\|{\tilde{x}}_{c,m,st,k,new}{\|}^{2}=\|\left(I_{J_{x}}-P_{c,m,st}{R_{c,m,st}}^{T}\right)x_{c,m,st,k,new}{\|}^{2} \end{array}$$

When new process data is obtained, the corresponding *T*^2^ and *SPE* values are calculated and compared with the control limits. If the values calculated from the new data are all below the control limit, the online batch process is considered to be normal. If the statistic calculated from the new data exceeds the detection limit at some point, the batch process production is considered to have failed and an alarm is issued.

For transition part data, the strategy of calculating monitoring statistics for the data at the *k*-th time is adopted one by one, based on the time slice method mentioned earlier. The model is modeled according to the following formulas. First, substitute data matrix at time slide *k* of transition part, $X_{c,m,tr,k}$, into the PLS model:

(16) $$\begin{array}{c} X_{c,m,tr,k}=T_{c,m,tr,k}{P_{c,m,tr,k}}^{T}+E_{c,m,tr,k}=\sum_{a=1}^{A}t_{c,m,tr,a,k}p_{c,m,tr,a,k}^{T}+E_{c,m,tr,k}\\ Y_{c,m,tr,k}=U_{c,m,tr,k}{Q_{c,m,tr,k}}^{T}+F_{c,m,tr,k}=\sum_{a=1}^{A}u_{c,m,tr,a,k}q_{c,m,tr,a,k}^{T}+F_{c,m,tr,k} \end{array}$$

Then, the monitoring statistics are calculated:

(17) $$\begin{array}{c} T_{c,m,tr,k}^{2}=x_{c,m,tr,k}^{T}R_{c,m,tr,k}{\left(\frac{{T_{c,m,tr,k}}^{T}T_{c,m,tr,k}}{I_{m}-1}\right)}^{-1}{R_{c,m,tr,k}}^{T}x_{c,m,tr,k}\\ SPE_{c,m,tr,k}=\|{\tilde{x}}_{c,m,tr,k}{\|}^{2}=\|\left(I_{J_{x}}-P_{c,m,tr,k}{R_{c,m,tr,k}}^{T}\right)x_{c,m,tr,k}{\|}^{2} \end{array}$$

where ${\tilde{x}}_{c,m,tr,k}$ is the residual matrix of time slide *k*, and $R_{c,m,tr,k}$ is calculated as follows:

(18) $$R_{c,m,tr,k}=W_{c,m,tr,k}{\left(P_{c,m,tr,k}^{T}W_{c,m,tr,k}\right)}^{-1}$$

$W_{c,m,tr,k}$ is the mass matrix. The corresponding control limits are as follows:

(19) $$\begin{array}{c} T_{c,m,tr,\alpha ,k}^{2}=\frac{H\left(I^{2}-1\right)}{I\left(I-H\right)}F_{c,m,tr,\alpha ,k}\left(H,I-H\right)\\ SPE_{c,m,tr,\alpha ,k}=g_{c,m,tr,k}\chi_{c,m,tr,h,\alpha ,k}^{2} \end{array}$$

After obtaining the data to monitor, $x_{c,m,tr,k,new}$, calculate the online *T*^2^ data and the online *SPE* data.

(20) $$\begin{array}{c} T_{c,m,tr,k}^{2}=x_{c,m,tr,k}^{T}R_{c,m,tr}{\left(\frac{{T_{c,m,tr}}^{T}T_{c,m,tr}}{I_{m}-1}\right)}^{-1}{R_{c,m,tr}}^{T}x_{c,m,tr,k}\\ SPE_{c,m,tr,k}=\|{\tilde{x}}_{c,m,tr,k}{\|}^{2}=\|\left(I_{J_{x}}-P_{c,m,tr}{R_{c,m,tr}}^{T}\right)x_{c,m,tr,k}{\|}^{2} \end{array}$$

The specific strategies for interpreting variables in the formula and online monitoring are the same as in a steady part.

## 3. Illustration and Discussion

### 3.1. Injection Molding Process

**Figure 6:**
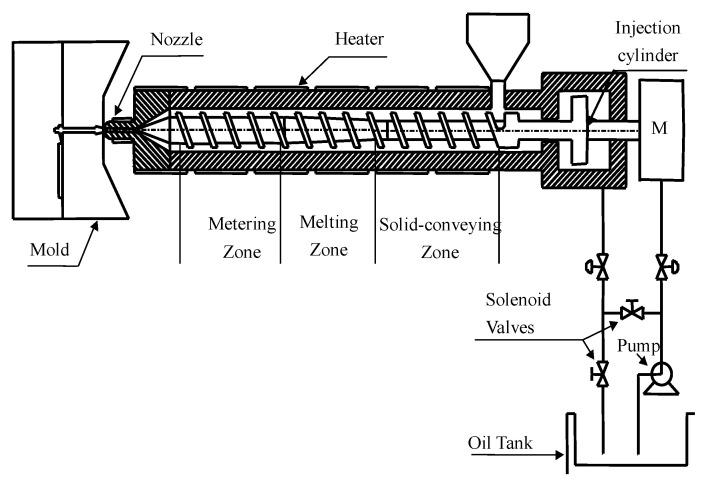
A simplified schematic diagram of the injection molding machine.

The injection molding process, which refers to the process of making semi-finished parts of a certain shape from molten raw materials through some specific operations, is taken as an example to illustrate the algorithm proposed in this paper. From the perspective of affecting the quality of injection molding products, all measurement variables can be divided into four categories, including equipment level, raw material level, process variable level, and external and internal disturbances. Process variables can reveal the actual running state of the injection molding process. Typical process variables of injection molding include melt temperature, pressure distribution, melt injection volume, cavity pressure, etc. In addition, the production process is also affected by machine parameters and material parameters. These variables of different levels and properties are complicated and coupled together to determine the quality of the final product. A schematic diagram of the injection molding machine is shown in Figure 6.

### 3.2. Variable Analysis of Injection Molding Process and Experiment Condition

The proposed algorithm is applied to a real injection molding process to verify its rationality. Quality (G) is chosen as product quality information. The material used in this work is high-density polyethylene (HDPE). Six process variables and one quality variable, as shown in Table 1, are selected for modeling. Under this operating condition, a total of 100 normal batches with 919 sampling points, six process variables, and one quality variable in each batch are obtained. The offline modeling and online monitoring methods mentioned in Section 2 are applied to this actual injection molding process.

### 3.3. Phase Division and Within-Phase Division

First, the phase division and inner-phase division methods proposed in Section 2.2.1 are performed to complete the evolutionary analysis of the direction of time. According to our previous experience [21], the injection process is divided into four phases, namely the injection stage, pressure holding stage, plasticizing stage, and cooling stage. Corresponding time data are 1–220, 221–519, 520–729, 730–919, respectively, for the concerned process. Then, the stable and transition parts are divided by the inner-phase division strategy in the proposed algorithm. The trend of $diff_{\beta }$ change in the direction of time slice *K*, which stands for the gradient change of the regression coefficient β matrix based on PLS model. The PLS algorithm is a classic method for modeling batch process data, which is straightforward and provides a wealth of information for modeling. Based on the PLS algorithm, the ridge regression algorithm [28] and the robust regression algorithm [29], etc., have been developed. These algorithms improve some of the shortcomings of PLS, but the complexity is higher. To verify the aptness of PLS in dealing with the problem of the batch process modeling in the article than the other algorithms, the ridge regression algorithm is taken as an example. The PLS algorithm and the ridge regression algorithm are used to complete the tasks of the inner-phase division, respectively, and the ability of the two algorithms to deal with batch process problems is compared. 

**Figure 7:**
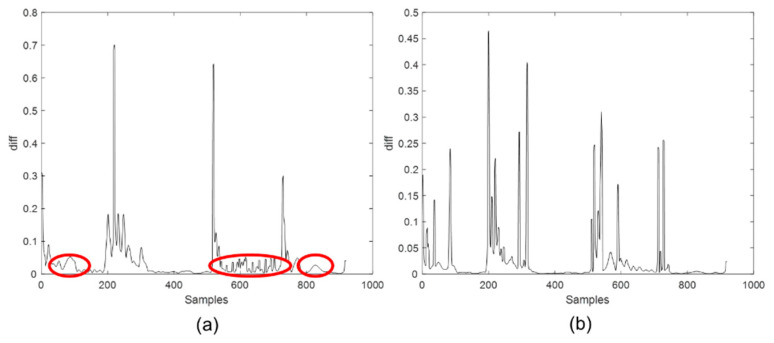
The trend of $diff_{\beta }$ calculated by (**a**) ridge regression method and (**b**) PLS method in the time direction.

Figure 7 shows the variation of the difference *diff* of the regression coefficients based on the ridge regression method with the sampling time and the change of the difference *diff* of the regression coefficients based on the PLS method with the sampling time. For the convenience of comparison, the data processing steps are completely consistent. The detailed method of these calculations is shown in Section 2.2.1. 

It can be seen that in the same experimental environment, the *diff* change calculated by the ridge regression algorithm has obvious instability in the first phase, the third phase, and the fourth phase. This is inconsistent with our previous knowledge of the injection molding process. Clearly, the ridge regression method amplifies the instability information, interfering with our inner-phase division task. Thus, the ridge regression method is difficult to achieve the task of division within phases. Although many improved methods of PLS, such as the ridge regression method, mainly overcomes the shortcoming of the insufficient regression ability of PLS for abnormal points, in the study of offline modeling of normal processes, there are no strict requirements on the processing ability of abnormal points. Therefore, the classical PLS algorithm is completely sufficient. The PLS algorithm is easy to implement and can provide a lot of variable information, such as score matrix, load matrix, etc., which are very important for capturing the evolution information in the direction of batch or time slice. If other algorithms are used, it will fail to reflect the advantages of the new algorithm and bring other risks. Therefore, as a well-recognized classical algorithm for state monitoring of batch processes, PLS is obviously applicable to research in this field. In addition, the main work of this article is not on the innovation of the PLS algorithm, and PLS is just a tool to realize innovative ideas. Therefore, the above discussion of the rationality of the PLS algorithm is completely sufficient. PLS will be used in the following experiments to achieve offline model establishment and online monitoring following the proposed strategy in Section 2. 

For the inner-phase division in the direction of time, it is worth mentioning that, after researching, it is found that within a reasonable threshold range, there is little difference in the results of inner-phase division for each time slice. As in the direction of time slice *K*, in the transition part, the variation of the regression coefficient β matrix is much larger than that in the steady part. That is, there is an obvious difference between the two, so it only needs to select a threshold within a reasonable range. For the injection molding process of this experiment, the threshold value of the first, second, and fourth phase is 0.005, and the threshold value of the third phase is 0.015, the parameter *m* in Equation (6) is set to 4.

**Figure 8:**
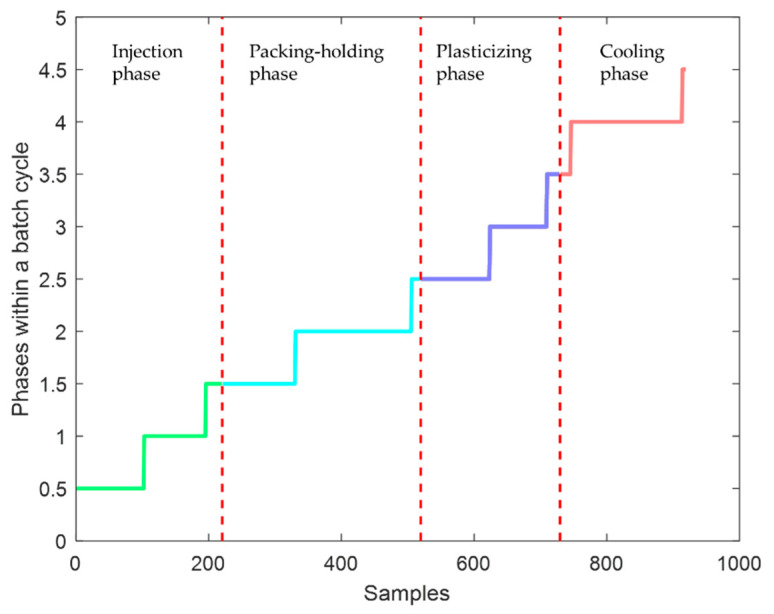
The final division results in the time direction.

As is shown in Figure 7b, the third phase is more volatile, which is related to the characteristics of the third phase of the injection molding process. The third phase, which belongs to the plasticizing phase, is also considered the beginning of the cooling stage, and the instability of the data reflects the freezing process of the mold gate [25]. In addition, in the region where the value of $diff_{\beta }$ is larger than the threshold value mostly, only a small part of the values is less than the threshold value. These few points are still classified into a transition part because the data before and after that time are still relatively unstable. The steady part and transition part of different phases are analyzed. The final division result in the time direction is shown in Figure 8. There are two transition parts in each phase, which are located before and after the steady part, respectively. These transition parts are considered as states between the two actual phases. In the injection phase, the steady part is 103~195; in the packing-holding phase, the steady time ranges from 331 to 505; and in the plasticizing phase, the steady time ranges from 624 to 709, while in the cooling phase, the steady time is 746~913. According to the classification results, it is easy to see that the steady part accounts for only about half of the whole phase in every phase, which means the influence of the transition part is not proposed to be ignored. The conclusion above proves the necessity of considering the steady and transition parts separately when modeling.

### 3.4. Mode Division Based on Sliding Windows

For each phase, the injection process’s evolution in the batch direction is studied using the sliding window model mentioned in Section 2.2.2.

**Figure 9:**
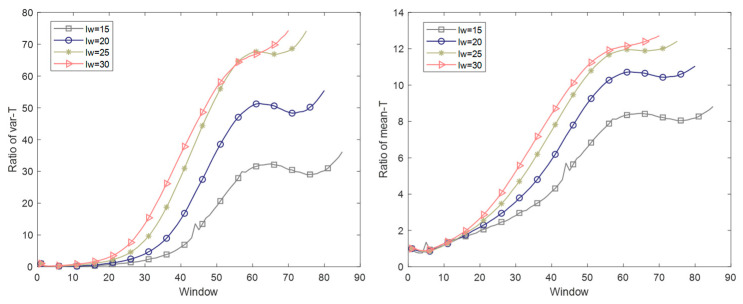
The variation trend of the score matrix with window length from 15 to 30.

The variation trend of the score matrix with window length from 15 to 30 and step of 5 is analyzed. The analysis applied in the first phase is shown in Figure 9, and results in other phases are proven to be similar. It can be seen from the result that both the *Ratio* calculated by the variance and mean of the score matrix **T** gradually increases with the window sliding down, and the increasing trend of the variance and mean is basically the same. Therefore, the following two judgments are made: 1. There is a slow evolution trend in the inter-batch score matrix **T**, which is reflected in gradually deviating from the first few batches; 2. The evolution trend of the mean and variance information of **T** changing along the sliding window is the same, but the magnitude of the increase is different. Based on the above judgment, it is necessary to carry out mode division in the sliding window batch direction. The mean value of **T** or the variance of **T** can be used as the standard for the mode division, and similar effects can be achieved. In addition, the influence of the window length on the evolution results is studied. When the window length is 15, some noise is not removed, but local information can be retained well. When the window length increases to 20 batches, the change rule of **T** is smoothed, and local information can also be retained well. With the further increase of the window length, the local data information is further smoothed and lost. As a result, the establishment of the sliding window loses its meaning. Therefore, the window size in the following is selected as 20 batches.

**Figure 10:**
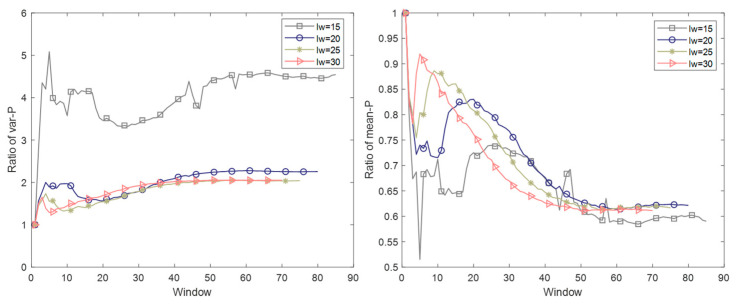
The variation trend of the load matrix with window length from 15 to 30.

In addition to the score matrix **T**, the evolution of the load matrix **P** in the batch direction is also studied. The result is shown in Figure 10. For the load matrix **P**, when the window length is 15, the instability is larger than the others, and the local variation information is obvious. With the increase of the window length, the instability decreases, and the curve becomes smoother. Therefore, the influence of the sliding window length parameter on modeling results can be reflected, and the operation rule of the sliding window can be revealed. Meanwhile, the regularity of the **P** index is not strong compared with that of **T**, so it is only used to study the influence of the window length on the modeling results, and the index **T** is used when dividing the modes.

**Figure 11:**
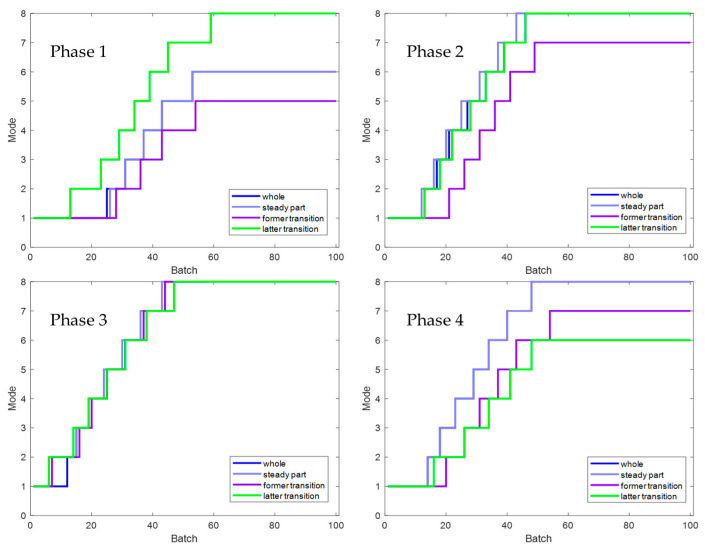
Mode division results in the batch direction of each phase.

Based on the above classification results of phases in the time direction, the data of former transition, steady part, and the latter transition are used respectively to build sliding windows and conduct mode division for them. Specifically, the mode division method mentioned in Section 2.2.2 is adopted; based on the variation rule of the variance of the score matrix **T**, the mode division in the batch direction is completed. As mentioned in Section 2.3, the mode to which the corresponding window belongs is the mode of this batch. In addition, except for the last few batches, in the experiment, batches, and windows with the same sequence number are considered to have a corresponding relationship. The result is shown in Figure 11. The result of mode division using the whole phase process data is also drawn simultaneously for comparison. The partition threshold selected in the experiment is 2.

From Figure 11, the following conclusions can be drawn. For the data of former transition, steady part and later transition, the results of mode division in the batch direction are different. In some phases, the mode division results completed by using transition part data have a big difference from those using the data of the entire phase, such as the 1st and 4th phases. The results of mode division using steady part data are very close to the results of mode division using the data of the entire phase, such as the 2nd and 3rd phases. This is because, in these phases, the transition part accounts for a smaller proportion, while the steady part accounts for a larger proportion, the steady part better represents the whole phase. These differences in mode division indicate that the strategy to model the transition part separately from the steady part is necessary. Analyzing the results in the graph from the perspective of batch evolution, it can be concluded that the first mode and the last mode contain a large number of batches. In contrast, the mode update is more frequent in the middle of the operation process, and each mode only contains about five batches. Such results can be explained by the operational characteristics of the injection molding process. The inter-batch variable evolution is relatively flat at the beginning and end of the injection molding process, so only a few modes need to be divided. In contrast, the batch evolution in the middle is faster, so the modes need to be updated frequently. Therefore, the mode division method proposed above captures the differences in information between batches in a more detailed manner, making the model more accurate, thus proving the rationality of modeling.

### 3.5. Online Monitoring Results and Discussion

The second phase, the packing-holding phase, is taken as an example to verify the strategy for online monitoring proposed in Section 2.3. To verify the universality of the algorithm, three batches of data are selected from 100 known batches of data collected, namely batches 11, 41, and 71, as test data, which represent the initial state, intermediate state, and end state of the batch operation process. The remaining data are modeled as training data. The following is a specific discussion.

Within the 11th batch, for the newly sampled variable data, it is first determined whether it belongs to the steady or transition parts. Then the modes are identified separately to calculate *T*^2^ and *SPE*. If the incoming data belongs to the former transition of the 11th batch, then it belongs to the first mode in the batch direction; that is, the mode is composed of batches 1–20. Therefore, the process data of the former transition and the first mode in the second phase are used for offline modeling. Similarly, if the incoming data belongs to the steady part, then it belongs to the first mode in the direction of the batch; that is, the mode is composed of batches 1–11. Therefore, the steady part process data of the first mode in the second phase are used for modeling. If the incoming data belongs to the latter transition, then it belongs to the first mode in the batch direction, the mode composed of batches 1–12. Therefore, the process data of the latter transition and the first mode in the second phase are used for modeling. Judged by the same method, the former transition data sampled from the 41st batch belongs to the sixth mode composed of batches 41–48 in the direction of the batch. However, the number of batches in this mode is too small to meet the modeling requirements; as mentioned in Section 2.2.2, the modeling data needs to be expanded; that is, the data of batches 49–58 need to be added for modeling. In other words, the process data of the former transition and the extended fourth mode in the second phase are used for modeling. For the steady part and the latter transition of the 41st batches, and the sample data of the 71st batches, the way of online monitoring is the same. The details are summarized in Table 2.

As shown in Table 2, for the same batch, the final attribution modes of samples in different parts of the phase may not be the same, which also verifies the importance of inner-phase division in the time direction. Secondly, as for whether to add more batches to meet the modeling requirements, there is no need to add batch training data for batches in the initial and the final batches, which indicates that most batches in the initial state and the final state belong to the same mode. While for intermediate batches, updates of mode are more frequent. This corresponds to the slow-fast-slow evolution trend of score matrix **T** previously drawn, which better verifies the rationality and necessity of modal division.

**Figure 12:**
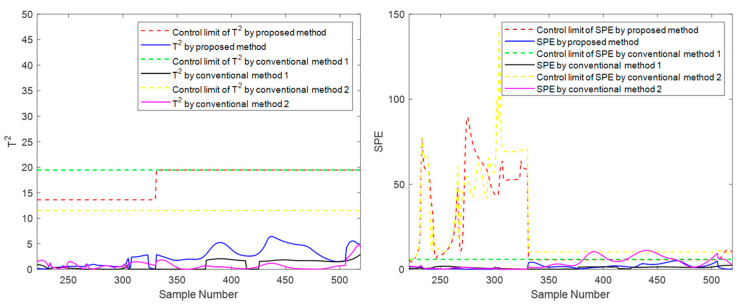
Online monitoring results of batch 11 by the proposed and conventional methods for the second phase.

**Figure 13:**
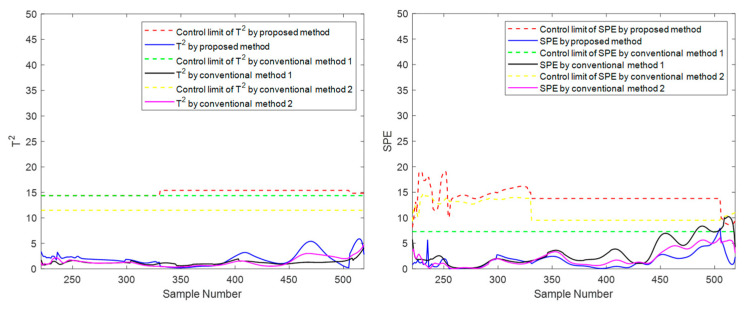
Online monitoring results of batch 41 by the proposed and conventional methods for the second phase.

**Figure 14:**
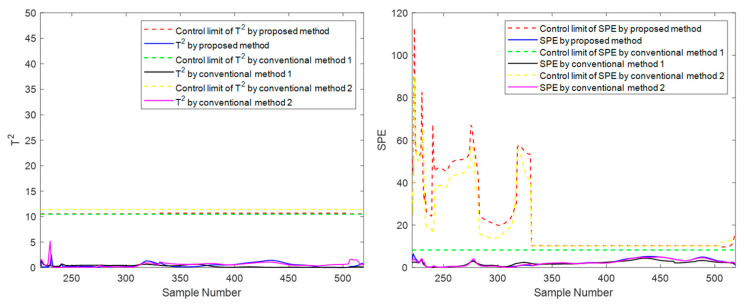
Online monitoring results of batch 71 by the proposed and conventional methods for the second phase.

In addition to the suggested method, two other traditional methods, conventional method 1 and conventional method 2, are set up in the experiment for comparison. Here is a brief introduction to both. Conventional method 1 uses the evolution information of the **T** index to carry out mode division in the direction of the sliding window, and its strategy and parameter settings are the same as the proposed method. Still, this method only carries out simple phase division in the time direction, ignoring the inner-phase’s differences and evolutionary laws. Compared with the proposed method, traditional method 1 has the same inter-batch evolution analysis strategy but a rough inner-batch evolution analysis strategy. Conventional method 2 divides the phases in the time direction and divides within-phase parts, separating the steady part and transition part for modeling. The strategy and parameter settings are the same as the proposed method. Still, it does not divide the modes in the batch direction using the evolution information but divides the modes with a fixed number of batches. Compared with the proposed method, traditional method 2 has the same inner-batch evolution analysis strategy but a rough inter-batch evolution analysis strategy. To meet the basic rationality of the method, batches are roughly considered to belong to three modes in equal order, representing the initial, intermediate, and final mode, respectively. Specifically, batches 1 to 33 are considered to belong to the initial mode, batches 34 to 66 are considered to belong to the intermediate mode, and batches 67 to 100 are considered to belong to the final mode. Batches 11, 41, and 71 selected as test batches in the experiment correspond to these three modes, respectively. These two traditional methods correspond to one-dimensional evolution information that only considers batch direction or time direction. The *T*^2^ and *SPE* indicators and their corresponding control limits are shown in Figure 12, Figure 13 and Figure 14.

It can be seen from Figure 12, Figure 13 and Figure 14 that the three methods can meet the requirements of online monitoring, among which the proposed method performs best, and both of the two traditional methods have the risk of model mismatch. Especially in batch 41, conventional method 1 exceeds the control limit a little in the later batches, which is deduced to be a frequent occurrence because the later batches are close to the transition part, and the data fluctuates greatly. This false alarm is easy to occur for conventional method 1, which makes no distinction between the steady part and the transition part. 

**Figure 15:**
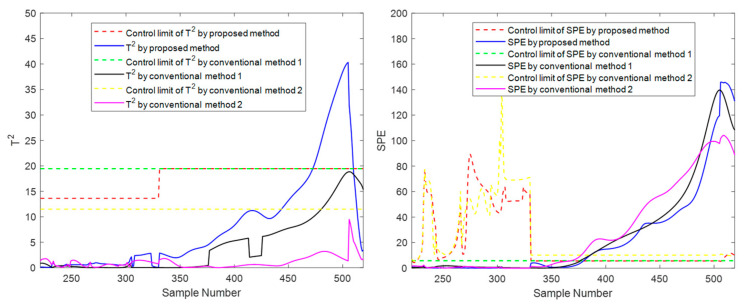
Online monitoring and fault diagnosis of batch 11 after introducing the fault.

**Figure 16:**
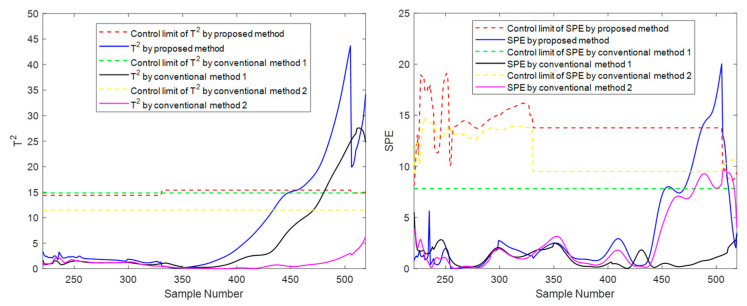
Online monitoring and fault diagnosis of batch 41 after introducing the fault.

**Figure 17:**
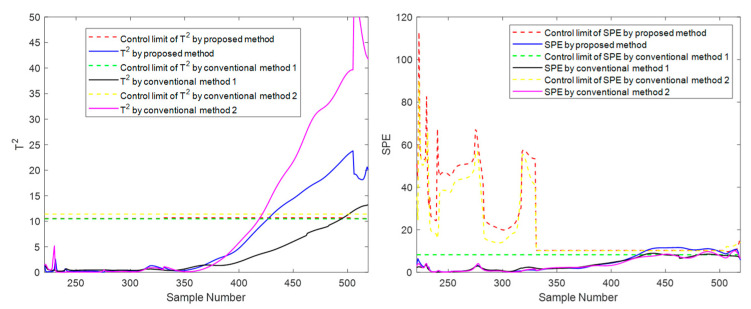
Online monitoring and fault diagnosis of batch 71 after introducing the fault.

At the moment 335, a slowly increasing failure is introduced for variable screw speed. The method proposed in this paper and the conventional methods are used for online monitoring. Batches 11, 41, and 71 are also selected for experimental investigation. The final online monitoring situation is shown in Figure 15, Figure 16 and Figure 17.

To compare the three methods more intuitively, the time when the *T*^2^ and *SPE* statistics exceed the control limit is listed in Table 3, which is used as the criterion to judge the sensitivity of the method to fault monitoring. Available in Table 3, for the slow fault, the proposed algorithm has obvious advantages on *T*^2^ statistic monitoring indicators, especially the initial state and the intermediate state. In batch 11, in the rest of the two traditional methods failed to detect fault cases, the proposed method of *T*^2^ index produces an alarm at time 473. In batch 41, the fault is detected at least 25 times faster than the proposed method’s traditional methods. This is sufficient to illustrate the rapidity of the proposed method; For *SPE* statistics, the proposed method is comparable to the traditional method with a slight advantage in robustness. For example, there is a serious lag in both conventional method 1 and conventional method 2 on batch 41 and a serious lag in conventional method 2 on batch 71. In addition, it can occasionally be seen from the table that the traditional two methods have a little advantage compared with the suggested method in some indexes of some batches. For example, in batch 71, conventional method 2 detected the fault five times faster than the suggested batch in the *T*^2^ index, but it is much inferior to the proposed method in other cases. There are even many cases of serious lag. Therefore, compared with conventional method 2, the proposed method is more robust on the whole and has higher sensitivity in most cases. The above results can be explained as follows: The two traditional methods that only consider one-dimensional evolution information are often not robust enough and have obvious shortcomings. As for conventional method 1, which only considers the evolution of batch direction, although it is sensitive to the slow time-varying information in batch direction, it is prone to miss diagnosis and misdiagnosis when dealing with different data of steady part and transition part in the time direction. For conventional method 2, which only considers changes in the time direction, the mode understanding in the direction of the batch is rough, and the fault monitoring is prone to lag and even model mismatch. The proposed method considers information in multiple dimensions and covers more comprehensive information, and can capture the change information in both directions, thereby providing a more appropriate monitoring strategy. It can be considered that the proposed method absorbs the advantages of the previous two conventional methods, studies the evolution information of time and batch in detail, and integrates them effectively, which can be more stable and effective in the case of a variable batch process.

## 4. Conclusions

This paper proposed a new monitoring strategy for batch processes, which more comprehensively captures the variable evolution information in the two dimensions of time and batch and establishes a more accurate online monitoring model based on these evolution laws. This method inherits some of the previous data modeling methods in the time direction and batch direction, using phase division, within-phase division in the time direction, and sliding window method in the batch direction, etc. Innovatively, those classical algorithms are improved, the operation mechanism of these methods is studied, and innovatively simpler processing is used to reduce the algorithm’s complexity. The biggest feature of this method is that the two-dimensional evolution information of time and batch is considered simultaneously, and the two are skillfully combined, showing superiority in both time and batch directions. Through experiments taking the injection molding process as an example, it can also be concluded that the method proposed in this paper overcomes the shortcomings of online monitoring caused by only considering one-dimensional evolution information and has stronger robustness and sensitivity. Moreover, it has obvious advantages, especially in the monitoring indicators of *T*^2^ statistics. Therefore, the algorithm proposed in this paper excels in online monitoring and fault diagnosis of batch processes and provides a new approach and research direction for high-performance monitoring of batch processes. 

## Figures and Tables

**Table 1:** Process and quality variables of an injection molding process.

Variable Type	Number	Variable Description	Unit
Process	1	Screw speed	Mm/s
	2	Plasticizing pressure	Bar
	3	Nozzle temperature	°C
	4	Cylinder pressure	Bar
	5	SV1 valve opening	%
	6	SV2 valve opening	%
Quality	1	Weight	g

**Table 2:** Specific arrangements for online monitoring of experimental batches.

Batch	Sub-Phase	Mode	Expansion
11	former transition	1	No
11	steady part	1	No
11	latter transition	1	No
41	former transition	6	Yes
41	steady part	7	Yes
41	latter transition	7	Yes
71	former transition	7	No
71	steady part	9	No
71	latter transition	8	No

**Table 3:** Comparison of fault monitoring capabilities of three methods.

Method	Batch	Time *T*^2^ Exceeding Limit	Time SPE Exceeding Limit
Proposed method	11	473	380
	41	457	487
	71	428	434
Conventional method 1	11	NAN	379
	41	482	NAN
	71	500	433
Conventional method 2	11	NAN	379
	41	NAN	NAN
	71	423	NAN

## Data Availability

The data presented in this study are openly available in https://share.weiyun.com/SwjfjW0Q, accessed on 6 March 2022.
